# Modeling sediment diagenesis processes on riverbed to better quantify aquatic carbon fluxes and stocks in a small watershed of the Mid-Atlantic region

**DOI:** 10.1186/s13021-020-00148-1

**Published:** 2020-07-06

**Authors:** Junyu Qi, Xuesong Zhang, Sangchul Lee, Yiping Wu, Glenn E. Moglen, Gregory W. McCarty

**Affiliations:** 1 Earth System Science Interdisciplinary Center, University of Maryland, College Park, 5825 University Research Ct, College Park, MD 20740 USA; 2 Joint Global Change Research Institute, Pacific Northwest National Laboratory and University of Maryland, College Park, MD 20740 USA; 3 Department of Environmental Science & Technology, University of Maryland, College Park, MD 20742 USA; 4 School of Human Settlements and Civil Engineering, Xi’an Jiaotong University, Xi’an, 710049 Shaanxi China; 5 USDA-ARS Hydrology and Remote Sensing Laboratory, Beltsville, MD 20705-2350 USA

**Keywords:** POC, DOC, Sediment diagenesis, Resuspension

## Abstract

**Background:**

Despite the widely recognized importance of aquatic processes for bridging gaps in the global carbon cycle, there is still a lack of understanding of the role of riverbed processes for carbon flows and stocks in aquatic environments. Here, we added a sediment diagenesis and sediment carbon (C) resuspension module into the SWAT-C model and tested it for simulating both particulate organic C (POC) and dissolved organic C (DOC) fluxes using 4 years of monthly observations (2014–2017) in the Tuckahoe watershed (TW) in the U.S. Mid-Atlantic region.

**Results:**

Sensitivity analyses show that parameters that regulate POC deposition in river networks are more sensitive than those that determine C resuspension from sediments. Further analyses indicate that allochthonous contributions to POC and DOC are about 36.6 and 46 kgC ha^−1^ year^−1^, respectively, while autochthonous contributions are less than 0.72 kgC ha^−1^ year^−1^ for both POC and DOC (less than 2% of allochthonous sources). The net deposition of POC on the riverbed (i.e., 11.4 kgC ha^−1^ year^−1^) retained ca. 31% of terrestrial inputs of POC. In addition, average annual buried C was 0.34 kgC ha^−1^ year^−1^, accounting for only 1% of terrestrial POC inputs or 3% of net POC deposition. The results indicate that about 79% of deposited organic C was converted to inorganic C (CH_4_ and CO_2_) in the sediment and eventually released into the overlying water column.

**Conclusion:**

This study serves as an exploratory study on estimation of C fluxes from terrestrial to aquatic environments at the watershed scale. We demonstrated capabilities of the SWAT-C model to simulate C cycling from uplands to riverine ecosystems and estimated C sinks and sources in aquatic environments. Overall, the results highlight the importance of including carbon cycle dynamics within the riverbed in order to accurately estimate aquatic carbon fluxes and stocks. The new capabilities of SWAT-C are expected to serve as a useful tool to account for those processes in watershed C balance assessment.

## Background

Recent studies highlight that carbon (C) cycling across terrestrial and aquatic environments are critical for bridging gaps in the global carbon cycle [1, 2]. The amount of C from terrestrial ecosystems exported to the oceans is only a fraction of that entering inland waters, and another fraction of this C is outgassed to the atmosphere as CO_2_ or is buried in freshwater sediments after erosion and transport from its sources [3, 4]. There are large uncertainties in estimations of C export from land, burial in water bodies, and outgassing from inland waters [5]. For instance, global estimates of terrestrial to aquatic C fluxes vary between 1.7 and 5.7 Pg C year^−1^ [6–8]; aquatic C burial is estimated to range from 0.15 to 1.6 Pg C year^−1^ [3, 9, 10]; and C outgassing estimates range from 0.75 to 3.88 Pg C year^−1^ [11–14]. Reducing uncertainties in estimates of aquatic C sources and sinks is critical for accurate quantification of the global C budget [15–18].

The aquatic C cycle in most watershed models is often oversimplified [19–24], though terrestrial C fluxes can be simulated with different levels of complexity of biogeochemical processes [25, 26]. In contrast, many water quality models require inputs from terrestrial processes to drive their simulations of complex aquatic processes such as hydrodynamics and biochemical conversion processes in streams and lakes [27–31]. In order to fill the gaps between watershed models and water quality models with respect to C cycling across terrestrial and aquatic ecosystems, we have developed terrestrial-aquatic C cycling algorithms within the framework of the Soil and Water Assessment Tool (SWAT) [24]. The enhanced SWAT model (hereafter SWAT-C) can simulate terrestrial C cycle processes including C uptake by photosynthesis, C release by plant and soil respiration, organic matter decomposition and mineralization, and disturbance processes (e.g., human activities) [32–34]. Furthermore, the model also represents aquatic C cycle processes including generation and transportation of total inorganic C (TIC), dissolved organic C (DOC), and particulate organic C (POC) from land to water bodies through runoff, leaching, erosion, and biogeochemical transformation processes between those different forms of C in freshwater [35, 36]. However, an important component of C fluxes in the aquatic environment, i.e., sediment diagenesis, was missing because there is a lack of representation of benthic carbon cycle processes within the SWAT-C model.

Particulate organic matter (including POC and algae debris) that is deposited onto the sediment bed will undergo complex decomposition and mineralization processes, which are referred to as sediment diagenesis [37]. The generated inorganic nutrients in the sediment on the riverbed can be recycled back to the water column via diffusion or resuspension resulting in an increased concentration of solutes. Nutrient resuspension is the process by which sediment porewater with elevated solute concentrations is mixed with the overlying water column due to sediment resuspension [38]. Deposited organic matter is also subject to sediment resuspension leading to accelerated mineralization rates in the overlying water column [39, 40]. In general, sediment is characterized as an sink of nutrients over years or decades; meanwhile sediment can become a net source by releasing previously-deposited nutrients to the water column over seasons or years [41, 42]. Nutrients released from the sediment and the amount of oxygen consumed during the process [i.e., sediment oxygen demand (SOD)] can contribute significantly to eutrophication and harmful hypoxia [43]. It is critical to model SOD and nutrient releases at the sediment–water interface in order to understand and mitigate the eutrophication problem and hypoxia in water bodies [28]. Therefore, for long-term simulations, an important aspect of water quality modeling is to describe sediment diagenesis processes and to estimate sediment fluxes released from the bed via diffusion and resuspension [43].

The aim of this research effort is to develop a benthic sediment diagenesis and C resuspension module, integrate it into SWAT-C, and apply it to understand the role of benthic carbon cycle processes in regulating aquatic carbon fluxes and stocks. Specifically, we conducted the following efforts: (1) developed a sediment diagenesis module within the frame-work of SWAT-C; (2) included nutrient resuspension processes in the sediment diagenesis module; (3) evaluated performance of the integrated model on POC and DOC simulations in a small watershed in the U.S. Mid-Atlantic region; (4) conducted parameter sensitivity analyses to identify important factors regulating coupled terrestrial-aquatic C cycling; and (5) analyzed various C fluxes associated with aquatic carbon cycling that include terrestrial inputs, buried bed sediments, outflow at the outlet, and deposition/resuspension on the riverbed.

## Data sources and methods

### Description of SWAT-C

The SWAT model is a continuous, physically-based, watershed-scale water quality model. It has been successfully employed and tested for simulating watershed hydrology, land surface water and heat exchange, and nutrient cycles across terrestrial and aquatic environments in a wide range of watersheds [44–49]. SWAT has been widely-used to simulate watershed water quantity and quality as affected by land use practices and climate change [50–57]. Recently, the CENTURY model [58] has been added to the SWAT-C model [32–34] to better refine the depiction of dynamics of soil organic matter (SOM) and residues including addition, decomposition, transformation, and removal of each SOM-residue pool present in surface and subsurface soil layers [32]. Further development of the SWAT-C model included new DOC/POC modules to simulate DOC/POC generation and transport processes in terrestrial environments [33, 35], and DOC/POC cycling in river networks [35, 36]. SWAT-C was successfully tested in the Cannonsville watershed in upper New York for simulating DOC fluxes [35], and two small watersheds in the Chesapeake Bay for POC fluxes [36]. In general, the current DOC/POC modules in SWAT-C allow it to satisfactorily estimate overall soil DOC/POC production and transport to streams, and to reproduce DOC/POC fluxes at the watershed outlet [35, 36]. Detailed model development and evaluation can be found in related publications [32, 33, 35, 36].

### Adding sediment diagenesis and sediment C resuspension processes into the SWAT-C model

**Fig. 1:**
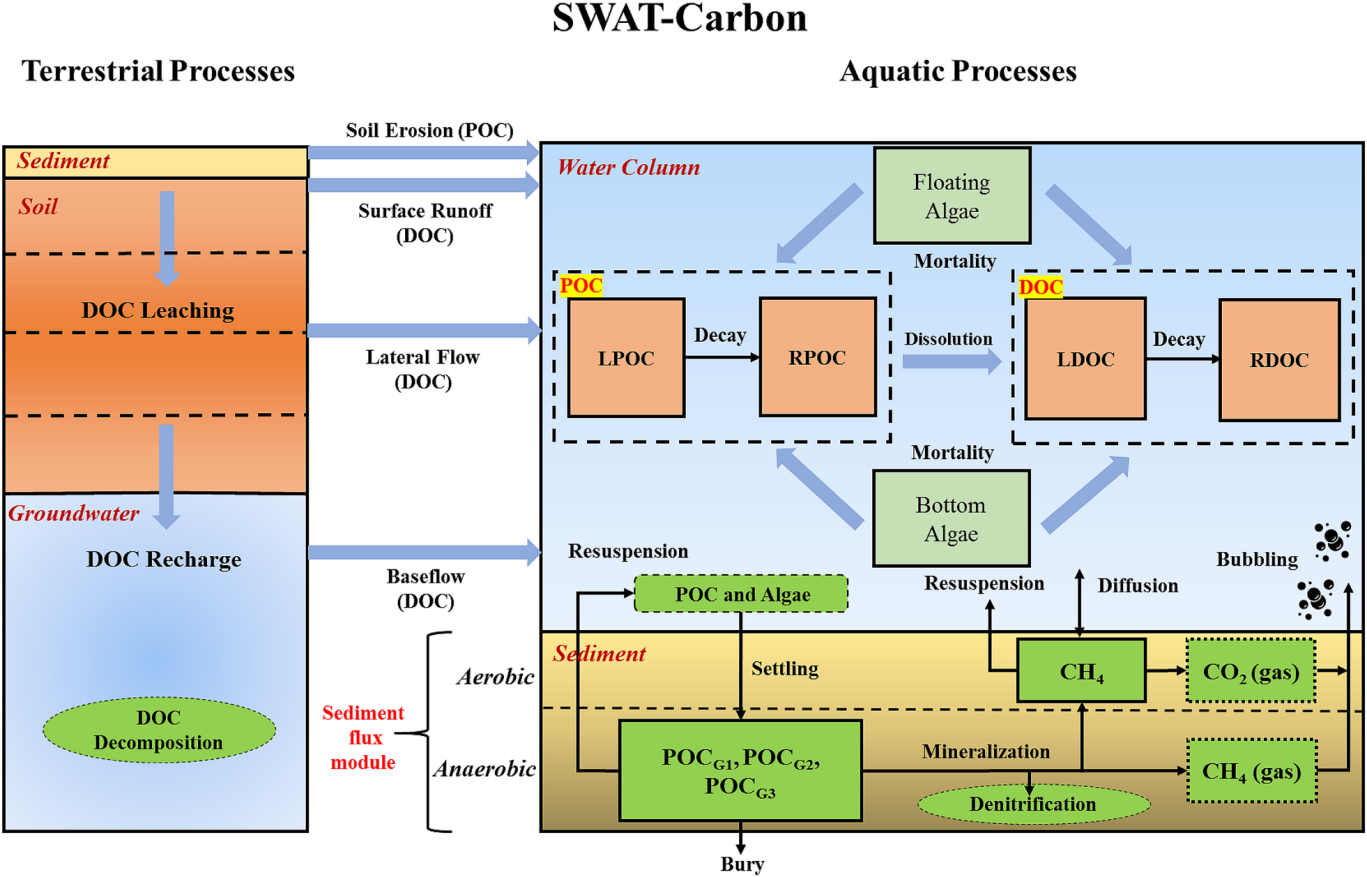
Simulated DOC and POC processes from terrestrial to aquatic environments within the SWAT-C model. The newly added sediment diagenesis and C resuspension processes are depicted at the sediment–water interface

A sediment diagenesis module was added to SWAT-C to depict the C fluxes between the river water column and the sediment bed. Sediment C fluxes are based on a model developed by Di Toro [59] and employed by QUAL2K [37, 41, 60]. Here, we further developed the sediment C resuspension process coupled with a sediment diagenesis model. A schematic of the sediment diagenesis and sediment C resuspension module coupled with DOC/POC modules in SWAT-C is depicted in Fig. 1. In the newly added sediment diagenesis model, sediments are divided into two layers: a thin (1 mm) surface aerobic layer underlain by a thicker (10 cm) lower anaerobic layer (Fig. 1). Organic C is delivered to the anaerobic sediments via the settling of particulate organic matter (i.e., floating algae and POC). The settled C is further categorized into three reactive fractions: labile (G1), slow reacting (G2) and non-reacting (G3). G1 and G2 fractions of settled organic C are subject to mineralization reactions. The mineralized organic C, after consumption by denitrification, is transformed into dissolved methane (CH_4_) in anaerobic sediments by the process of methanogenesis. Because methane is relatively insoluble, its saturation can be exceeded, and CH_4_ gas (as bubbles) may be produced in anaerobic sediments (Fig. 1). Dissolved CH_4_ are then transported to the aerobic layer where some of the CH_4_ is oxidized into CO_2_ and the remaining dissolved CH_4_ is transported to the overlying water column via diffusion. Generated CO_2_ gas (as bubbles) in the aerobic sediment and CH_4_ gas (as bubbles) in the anaerobic sediment are lost from the sediment by bubbling processes into the overlying water column. Labile (G1), slow reacting (G2), non-reacting (G3) settled organic C and dissolved CH_4_ are subject to resuspension processes (Fig. 1). A fraction of settled organic C is buried out of the sediment system. Mass balance for organic C and CH_4_ in the sediment is illustrated in Fig. 1.

The mass balance for labile (G1), slow reacting (G2), and non-reacting (G3) organic C in the anaerobic sediment layer considering C resuspension is written as [37],

1 $$H_{2} \frac{{dPOC_{Gi} }}{dt} = J_{POC,Gi} - J_{{POC_{R} ,Gi}} - J_{{POC_{MIN} ,Gi}} - J_{{POC_{Bury} ,Gi}}$$

where *POC*_*Gi*_ is the concentration of the Gi (i = 1, 2, or 3) organic C in the anaerobic sediment layer (g m^−3^), *H*_*2*_ is the thickness of the anaerobic layer (= 0.1 m), *dt* is the time step (day), $J_{POC,Gi}$ is the flux of Gi organic C delivered to the anaerobic layer (g m^−2^ day^−1^), $J_{{POC_{R} ,Gi}}$ is the resuspension flux for Gi organic C (g m^−2^ day^−1^), $J_{{POC_{MIN} ,Gi}}$ is the mineralization flux (g m^−2^ day^−1^), and $J_{{POC_{Bury} ,Gi}}$ is the burial flux for Gi organic C (g m^−2^ day^−1^). Organic C resuspended from the sediment bed is calculated by [43],

2 $$J_{{POC_{R} ,Gi}} = \frac{{POC_{Gi} }}{{\rho_{sed} \cdot 1000000}} \cdot J_{SED\_R}$$

where $\rho_{sed}$ is sediment density (Mg m^−3^), and *J*_*SED_R*_ is the resuspended sediment flux (g m^−2^ day^−1^), which is calculated with the sediment deposition and resuspension algorithm in SWAT.

A CH_4_ mass balance can then be written for the aerobic layer as [37],

3 $$H_{1} \frac{{dCH_{4} }}{dt} = J_{CH4,d} - J_{CH4,w} - J_{CH4,R} - J_{CH4,o}$$

where *CH*_*4*_ is the methane concentration in the aerobic layer (g m^−3^), *H*_*1*_ is the thickness of the aerobic layer (= 0.001 m), $J_{CH4,d}$ is the flux of dissolved CH_4_ that is generated in the anaerobic layer and delivered to the aerobic sediment (g m^−2^ day^−1^),$J_{CH4,w}$ is the CH_4_ diffusion flux to the overlying water column (g m^−2^ day^−1^), $J_{CH4,R}$ is the CH_4_ resuspension flux (g m^−2^ day^−1^), and $J_{CH4,o}$ is the CH_4_ oxidation flux (CO_2_ gas as the product) in the aerobic sediment (g m^−2^ day^−1^). CH_4_ resuspended from the sediment bed is calculated by [43],

4 $$J_{CH4,R} = \frac{{CH_{4} }}{{\rho_{sed} \cdot 1000000}} \cdot J_{SED\_R}$$

The $J_{CH4,d}$ is calculated in the anaerobic layer as [37],

5 $$J_{CH4,d} = J_{C} - J_{C,den} - J_{CH4,gas}$$

where $$J_{C}$$ is the C diagenesis flux (g m^−2^ day^−1^; = $$J_{{POC_{MIN} ,G1}} + J_{{POC_{MIN} ,G2}} )$$, $$J_{C,den}$$ is the C flux consumed by denitrification (g m^−2^ day^−1^), $$J_{CH4,gas}$$ is the CH_4_ gas flux generated in the anaerobic layer when $$J_{C}$$ is sufficiently large (g m^−2^ day^−1^). Detailed information regarding the calculation of various C fluxes in the aerobic and anaerobic layers can be found in [37]. It is worth noting that default values were adopted in the present study for all the coefficients and transformation rates used for associated sediment diagenesis flux calculations [37].

### SWAT-C parameterization

Within the SWAT-C model, Du et al. [35] identified six calibration parameters controlling DOC cycling. Qi et al. [36] further sorted out eight calibration parameters for POC cycling. Based on the results from Qi et al. [36], two DOC and three POC associated parameters were identified as the most sensitive. The following five parameters were chosen to calibrate DOC and POC cycling: the DOC percolation coefficient (*β*_*DOC*_) which specifies the concentration of DOC in surface runoff as a fraction of the concentration in percolation; the liquid–solid partition coefficient (*k*_*OC*_) which determines the production of DOC in soil solution; the POC enrichment ratio (*ER*_*POC*_) which is defined as the ratio of the concentration of POC in eroded soils to the concentration of soil organic C in the soil surface layer; and the LPOC and RPOC settling velocity (*V*_*lpoc*_ and *V*_*rpoc*_, respectively) which control the deposition of POC (Table 1).

**Table 1:** Calibrated model parameter values for flow rate and sediment, POC and DOC loads in the study watershed

Variable	Parameter*	Explanation	Calibrated value
	V_SFTMP	Snowfall temperature (°C)	− 0.375
	R_CN_2_	Curve number	−9%
	V_SURLAG	Surface runoff lag coefficient	0.28
Flow rate	V_ESCO	Soil evaporation compensation factor	0.77
	V_SLSOIL	Slope length for lateral flow (m)	13.5
	V_ALPHA_BF	Baseflow alpha factor (1/days)	0.0565
	V_GW_DELAY	Groundwater delay (day)	13.5
Sediment	R_USLE_K	USLE soil erosivity factor	0
	V_USLE_P	USLE support practice factor	1
	V_ADJ_PKR	Peak rate adjustment factor in tributary channels	1
	V_PRF	Peak rate adjustment factor in main channels	0.022
	V_SPCON	Linear parameter for sediment routing in main channels	0.003238
	V_SPEXP	Exponent parameter for sediment routing in main channels	1.1975
	V_*ER*_*POC*_	POC enrichment ratio	2.98
POC	V_*V*_*lpoc*_	LPOC settling velocity (m day^−1^)	0.12
	V_*V*_*rpoc*_	RPOC settling velocity (m day^−1^)	0.36
DOC	V_*k*_*OC*_	Organic C partition coefficient	4185
	V_*β*_*DOC*_	DOC percolation coefficient for top soil	0.83

*The leading letters R and V in the parameters names stand for relative change (%) and replace the default value with the adjusted value [97], respectively

### Study area and data collection

**Fig. 2:**
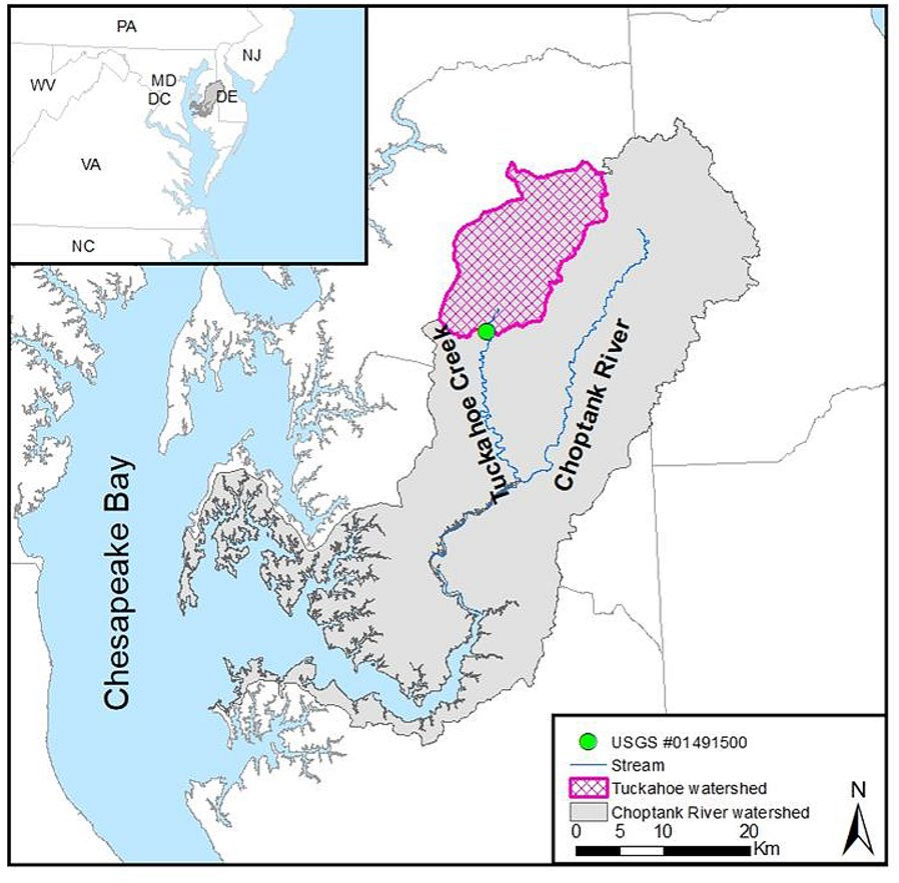
The location of Tuckahoe Watershed and USGS gauging station at the outlet of the watershed

We used a small watershed, i.e., the Tuckahoe Watershed (TW; 220.7 km^2^), defined by the United States Geological Survey gauging station (USGS#01491500) at Tuckahoe Creak near Ruthsburg, MD (Fig. 2), to simulate C fluxes from terrestrial to riverine ecosystems. The TW is located in the headwaters of the Choptank River Watershed (CRW) in the coastal plain of the Chesapeake Bay (Fig. 2). The major land uses in the TW are agriculture (54.0%) and forestry (32.8%), and it is dominated by well-drained soils (56.1%; Hydrologic Soil Group, HSG-A&B) [61]. The Othello soil series (fine-silty) and the Mattapex soil series (fine-silty) are two commonly found cropland soil types in the TW [62]. Topography within the TW is relatively flat, with most areas having less than 2% slopes. The study area is characterized by a temperate and humid climate with an average annual temperature of 15.4 °C and an average annual amount of ca. 1200 mm precipitation [63, 64].

We used a soil map based on the Soil Survey Geographic Database (SSURGO) from the United States Department of Agriculture (USDA), Natural Resources Conservation Service (NRCS) to provide soil properties information, and a 10-m Light Detection and Ranging (LiDAR)-based Digital Elevation Model (DEM) to provide topographic information. At the same time, the scheduling of crop rotations was generated using 2008–2012 data from the USDA-National Agriculture Statistics Service (NASS) Cropland Data Layer (CDL). High resolution (~ 1/8°) National Astronautics and Space Administration (NASA) North-American Land Data Assimilation System 2 (NLDAS2) climate forcing data [65] were used to provide daily weather inputs including precipitation, temperature, solar radiation, relative humidity, and wind speed [66]. For more information regarding SWAT model setup in the TW, please refer to Lee, Yeo [61].

We conducted monitoring of riverine total organic C (TOC) and DOC concentrations at the Ruthsburg USGS gauging station for the TW (Fig. 2). The in situ instrument packages containing full spectrum (200 to 700 nm) spectrophotometer probes (S-CAN Instruments; Vienna Austria) which perform water quality sampling at 30-min intervals were used. Multiwavelength calibrations were used for TOC and DOC in the stream water [67]. Fine time-resolution TOC and DOC data were aggregated to a monthly time scale for SWAT-C calibration and validation. POC was derived by subtracting DOC from TOC. Sediment data were collected from the United States Environmental Protection Agency (USEPA) Chesapeake Bay Program (CBP) water quality database [68]. In total, there are only 37 sediment concentration data from 2014 to 2017. We calculated daily sediment load by multiplying sediment concentration by stream flow of the sampling day.

### Model calibration, validation, and sensitivity analysis

The sampling period (Jan. 2014–Oct. 2017) was divided for calibration (2014–2015) and validation (2016–2017) purposes. Before the calibration period, we used a 2-year (2012–2013) warm-up period to initialize the SWAT-C model. We chose the most frequently calibrated parameters for monthly flow rate based on previous studies conducted in the study watersheds [36, 69]. We adopted a multi-step procedure provided by the previous study to calibrate POC and DOC loads [36]. The first step was to calibrate streamflow because DOC and POC loads are closely associated with water flow. After POC load calibration, DOC load is finally calibrated. In the present study, we used limited sediment data to calibrate sediment related parameters to reduce the uncertainty of predicting POC which is closely associate with sediment generation, transport, and deposition from the uplands to the watershed outlet.

We used the Sequential Uncertainty Fitting algorithm version 2 (SUFI-2) method in SWAT-CUP [70] to conduct auto-calibration for monthly flow rate and POC and DOC loads. We used the global sensitivity analysis method for sensitivity analysis. The global sensitivity analysis approach is a multiple regression system given as:

6 $${\text{g}} =\upalpha + \mathop \sum \limits_{i = 1}^{m} \beta_{i} \cdot b_{i}$$

where g is the objective function value, *α* and *β*_*i*_ are regression coefficients, *b*_*i*_ is the calibrated value of the *i*th parameter, and *m* is the number of parameters considered. We employed the Nash–Sutcliffe coefficient-NS [71] as the objective function (g = NS) because it is a commonly used goodness-of-fit coefficient in hydrologic modeling studies. Further, model performance evaluation criteria have been established by Moriasi, Arnold [72] for the NS metric. A student t-test was used to quantify the statistical significance of each parameter, with a p-value < 0.05 indicating a parameter as sensitive in the present study. We also provided parameter sensitivity rankings based on p-value for additional sensitivity analysis.

### Model performance evaluation

Two widely-used statistical criteria, i.e., percent bias (P_bias_) and NS, are used for model evaluation and given as:

7 $$P_{bias} = 100 \cdot \frac{{\left( {O_{avg} - P_{avg} } \right)}}{{O_{avg} }}$$

8 $$NS = 1 - \frac{{\mathop \sum \nolimits_{i = 1}^{n} (O_{i} - P_{i} )^{2} }}{{\mathop \sum \nolimits_{i = 1}^{n} (O_{i} - O_{avg} )^{2} }}$$

where *O*_*i*_ and *P*_*i*_ are the observed and predicted values, and *O*_*avg*_ and *P*_*avg*_ are the average of the observed and predicted values, respectively.

## Results and discussion

### Model performance and sensitive analysis

Calibrated parameter values with respect to the three water quantity and quality variables in the study watershed are shown in Table 1. Model performance for simulation of monthly flow rate and POC and DOC loads as indicated by NS and P_bias_ during calibration and validation are shown in Table 2. Because there is no established criteria for model performance evaluation for POC and DOC, we assumed the widely-accepted criteria for nitrogen (N) and phosphorus (P) loads from Moriasi, Arnold [72] were applicable to POC and DOC. As a result, model simulation can be judged as satisfactory if NS > 0.50 at a monthly time step, while − 25% < P_bias_ < 25% for streamflow and − 70% < P_bias_ < 70% for POC and DOC regardless of simulation time step [72].

**Table 2:** Model performance on monthly flow rate and POC and DOC loads in the study watershed

Variable	Calibration		Validation	
	NS	P_bias_ (%)	NS	P_bias_ (%)
Flow rate	0.79	5.6	0.86	6.1
POC	0.75	− 14.6	0.67	22.7
DOC	0.84	− 19.8	0.58	14.1

In general, the calibration results demonstrate satisfactory model performance for the three water quantity and quality variables based on two statistics during both calibration and validation (Table 2). Model performance for streamflow during validation was slightly better than that during calibration. In contrast, model performance for POC and DOC loads during calibration was slightly better than that during validation (Table 2). We also found that POC and DOC loads were underestimated during validation compared with calibration results (as indicated by positive P_bias_ values). This is understandable because only 2 years of water quality data were used for calibration which may not sufficiently account for different hydroclimatic and management conditions.

**Fig. 3:**
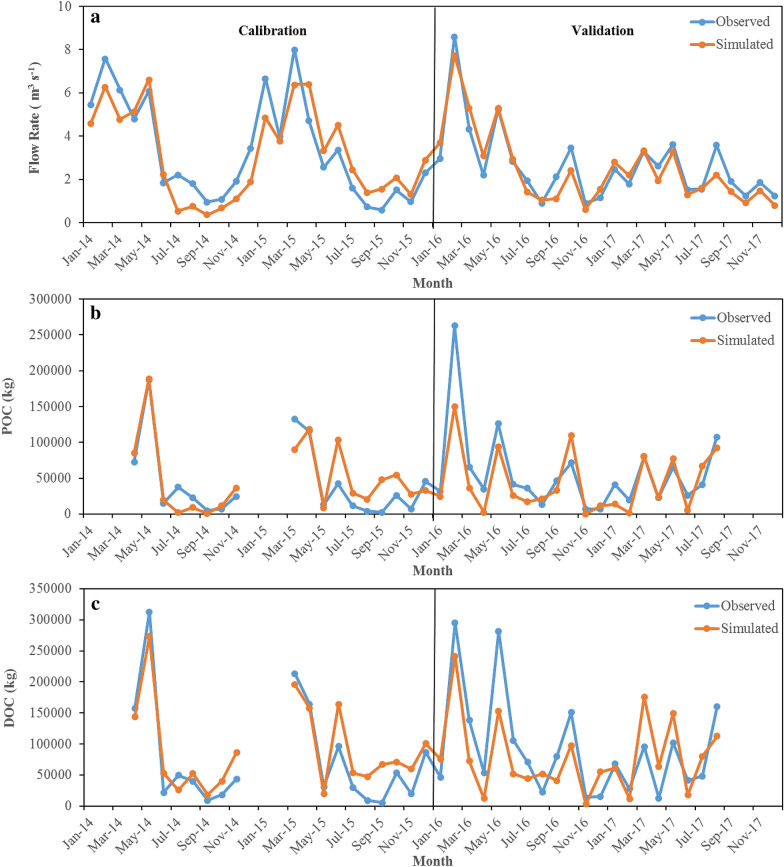
Simulated vs. observed monthly flow rate (**a**) and POC (**b**) and DOC (**c**) loads during calibration and validation in the Tuckahoe Watershed

Simulated vs. observed monthly flow rate and POC and DOC loads are shown in Fig. 3 during calibration and validation. In general, simulated monthly variation of streamflow and POC and DOC loads matched observations well. The results were consistent with the previous study conducted in the same watershed with only 2 years of measurement [36]. As that study pointed out, the SWAT-C model tended to underestimate some high flows which were dominated by surface runoff in the watershed. It has been widely reported that the SWAT model tends to underestimate surface runoff especially under wet conditions [73, 74]. Not surprisingly, peak DOC and POC loads were also underestimated (Fig. 3c) because of the dependency of DOC and POC fluxes on streamflow [35].

**Fig. 4:**
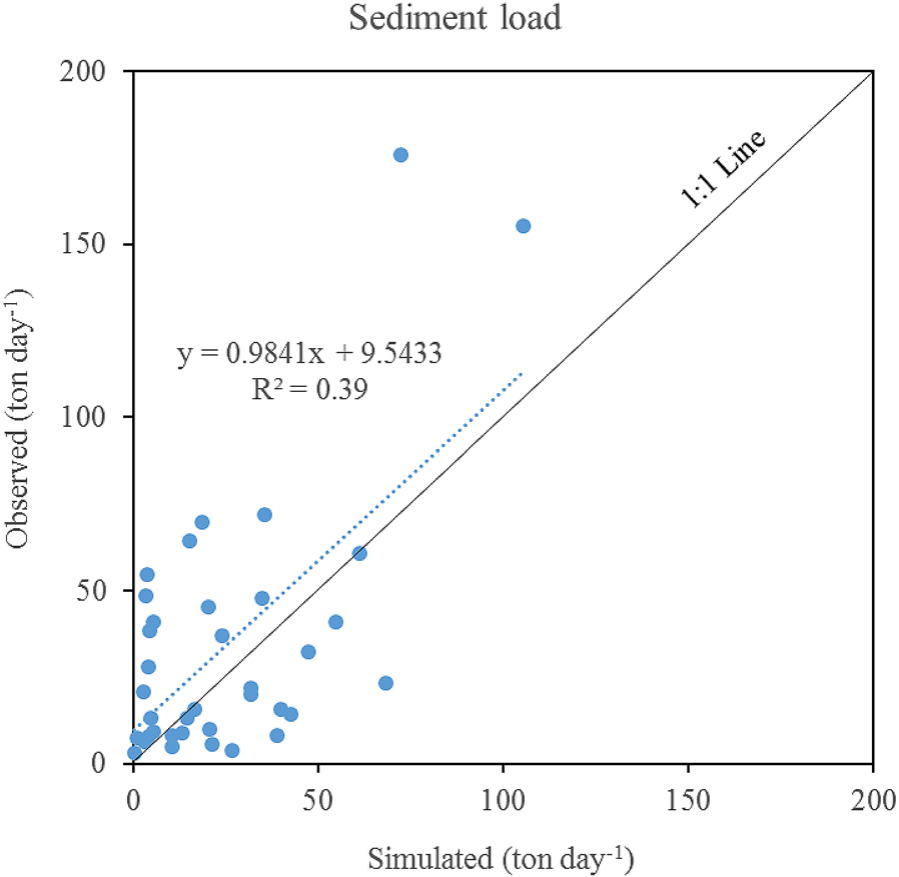
Simulated vs. observed daily sediment loads during 2014–2017 in the Tuckahoe Watershed (including 37 observed data points)

Since we only have 37 daily sediment load data during 2014-2017, we calibrated sediment-related parameters at the daily time step (Table 1), and the simulation vs. observation result is shown in Fig. 4. Note that we did not conduct calibration and validation for sediment in the same manner for flow rate, POC, and DOC because of limited data points. The simulated sediment loads explained about 40% of the variation in the observed data (R^2^ = 39%) and the regression line between simulation and observation was very close to 1:1 line indicating acceptable model performance (Fig. 4). The purpose of calibrating sediment-related parameters is to reduce POC prediction uncertainty mainly due to two major processes: (1) POC mobilization and transport from the uplands by erosion and (2) POC resuspension from sediments of riverbed. Sediment-related parameters controlling the former include USLE_K, USLE_P, and ADJ_PKR, and parameters controlling the latter include PRF, SPCON, and SPEXP (Table 1). Auto-calibration method (SUFI-2) was initially used to calibrate daily sediment loads by adjusting values of these six parameters. The calibrated values of USLE_K, USLE_P, and ADJ_PKR were significantly lower than default values indicating less sediment yields from the uplands. However, subsequent POC calibration generated unrealistic values of POC enrichment ratio (*ER*_*POC*_ > 3.5) and settling velocities for LPOC and RPOC (*V*_*lpoc*_ and *V*_*ppoc*_ < 0.01 m day^−1^) which indicates underestimation of terrestrial POC. As a result, we adopted default values for upland sediment-related parameters and only calibrated main channel parameters (Table 1). The result suggests that soil erosion processes have significant impacts on POC generation and transport on the uplands, and simultaneous calibration of sediment and POC are recommended.

Using the global sensitivity analysis approach only reveals the “partial truth” about parameter sensitivity since it provides relative sensitivity about certain parameters. However, the relative parameter sensitivity provides valuable information on the relative importance of different physical and biogeochemical processes within the whole system. This analysis improves our understanding of the model performance as well as the underlying physical processes. Thus, we conducted parameter sensitivity analyses to investigate the relative importance of POC settling and resuspension processes in the TW (Table 3). Three sediment deposition and resuspension parameters related to C resuspension processes in a reach segment are the peak rate adjustment factor (PRF) and the linear and exponential calibration parameters (SPCON and SPEXP, respectively). Sensitivity analysis results indicated that the two most sensitive parameters were *V*_*lpoc*_ and *V*_*rpoc*_ with p-values less than 0.05 indicating significant sensitivity to POC load, while other sediment-related parameters had insignificant impacts (with p-value > 0.05). The results suggest that parameters that regulate POC deposition are more sensitive than those determining POC resuspension from sediments indicating the dominance of POC settling processes in the watershed.

**Table 3:** Sensitivity analysis of POC deposition and C resuspension parameters to POC loads

Parameter	p-value	Ranking
*V*_*lpoc*_	0.000	1
*V*_*rpoc*_	0.000	2
SPCON	0.205	3
SPEXP	0.473	4
PRF	0.757	5

### Analyzing riverine POC and DOC fluxes and stocks

Averaged annual POC and DOC fluxes from terrestrial to aquatic environments (including fluxes from uplands, buried in sediments, outflow at the outlet, and deposition/resuspension) in the study watershed are summarized over 2014–2017 (Table 4). In general, POC in riverine environments mainly originates from two sources including erosion of soil organic carbon and autochthonous production, which vary with distance from the headwaters, land use types, and hydrological conditions [75]. For the TW, model simulated average annual POC fluxes derived from various land use types (allochthonous sources) was about 36.6 kgC ha^−1^ year^−1^ during 2014–2017 at the watershed scale. In comparison, POC fluxes originating from river systems (autochthonous sources) was about 0.05 kgC ha^−1^ year^−1^ which is negligible. These results are consistent with the conclusion that soil erosion plays a dominant role in POC export [76]. Although it was estimated that autochthonous production provides approximately 8–28% of POC in large rivers [77, 78], for a small headwater watershed such as the TW, it is reasonable that POC from allochthonous sources dominates.

**Table 4:** Average annual POC and DOC fluxes from terrestrial to aquatic environments in the study watershed summarized over 2014-2017

Variable	Watershed contri.	Riverine contri.	Outlet outflow	Deposition	Resuspension	Net deposition	Bury
POC (kgC ha^−1^)	36.6	0.05	24.5	11.44	0.04	11.4	0.34
DOC (kgC ha^−1^)	46	0.72	46.3	–	–	–	–

	Out/WC	NDep/WC	NDep/Out	Res/Dep	Bury/WC	Bury/Out	Bury/NDep
POC	0.67	0.31	0.47	0.004	0.009	0.014	0.03
DOC	1.01	–	–	–	–	–	–

The table also includes average annual deposition and resuspension amounts of POC over the entire watershed and ratios between fluxes for POC and DOC

Out, WC, Dep, Res, and NDep indicate outlet outflow, watershed contribution, deposition, resuspension, and net deposition (equal to deposition–resuspension), respectively

About 24.5 kgC ha^−1^ year^−1^ POC leaves the outlet of the TW, which accounts for 67% of total POC from the uplands to the rivers (Table 4). Net deposition (equal to deposition–resuspension) of POC on the sediment bed was about 11.4 kgC ha^−1^ year^−1^ which accounted for 31% of total POC coming from the uplands. More detailed breakdown numbers are: 11.44 kgC ha^−1^ year^−1^ POC deposited and 0.04 kgC ha^−1^ year^−1^ POC resuspended. The resuspended POC only accounted for 0.4% of total deposited POC in the watershed confirming the conclusion from sensitivity analysis that the POC settling rather than C resuspension is the dominating C cycling process in the TW. We also found that net deposition of POC represents 47% of POC fluxes exported from the watershed, indicating the very important role of POC deposition for aquatic carbon fluxes. Annual POC export from the watershed is also within the reported values for forested and agricultural watersheds from literatures [79–81]

For DOC, about 46 kgC ha^−1^ year^−1^ was exported annually from the uplands via surface and subsurface water flow to the watershed river system, and about 0.72 kgC ha^−1^ year^−1^ was generated within the river system, which is less than 2% of the amount generated from the uplands (Table 4). Simulation results indicate the majority of DOC loads originated from terrigenous sources in the study watershed which is consistent with conclusions from many other studies [82, 83]. This result agrees with POC and confirms the argument that the dominant source of DOC and POC to most rivers is of terrestrial origin [84]. Previous studies with similar climate to our study estimated 10 kgC ha^−1^ year^−1^ DOC export from a small forested watershed (12 ha) [81] and more than 22 kgC ha^−1^ year^−1^ DOC export (based on only two extreme storms within 1 year) from a small agricultural watershed (21 ha) [85], which are consistent with our estimated average annual DOC export from the TW which is a mixed land use watershed with about 54% agriculture lands and 33% forestry lands.

About 46.3 kgC ha^−1^ year^−1^ of DOC was transported to the outlet of the watershed. DOC could be lost by mineralization (DOC converted to DIC), and its concentration in the river system could increase as a result of algae mortality and POC dissolution (Fig. 1). Simulation results show that most DOC comes from uplands and indigenously generated DOC was lost from the watershed and caused marginal changes to DOC concentration during the study period. The estimated annual DOC export from the watershed is also within the range of 10 to 100 kgC ha^−1^ year^−1^ reported in a review of 40 catchments worldwide by Hope, Billett [86]. The higher ratio of POC to DOC fluxes at the outlet of the watershed compared with that from the uplands was mainly caused by net deposition of POC on the sediment bed. In addition, the result is consistent with the general conclusion that organic C exported from terrestrial to aquatic environments has a high percentage of DOC [84, 87].

### Estimation of burial C

Average annual burial C was estimated to be about 0.34 kgC ha^−1^ year^−1^ during 2014–2017 in the study watershed, which accounted for about 0.9% of POC fluxes from uplands, 1.4% of POC transported to the outlet, or 3% of net POC deposition, respectively (Table 4). Compared with studies in lakes and coastal environments, the annual C burial rates in this study watershed were considerably smaller [88–92]. One possible reason is that the value for burial rate used in the sediment diagenesis model is lower than those used in other studies. For example, the default burial velocity for CE-QUAL-W2 [30] is 0.001 m day^−1^ while the value adopted here is 6.85E−06 m day^−1^ [37]. Considering the large range of burial velocities reported in inland waters and coastal environments [93–95], we could expect large uncertainty regarding estimation of buried C at the watershed scale without observed data.

### Quantification of the benthic carbon cycle processes

**Fig. 5:**
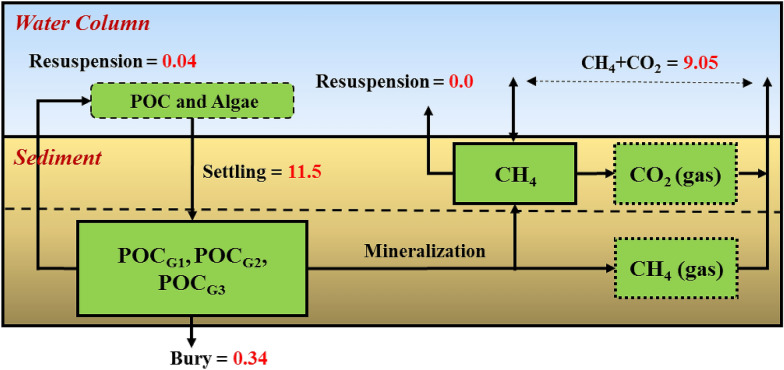
Benthic carbon cycle fluxes for the TW from 2014 to 2017. Unit: kg ha^−1^ year^−1^

**Fig. 6:**
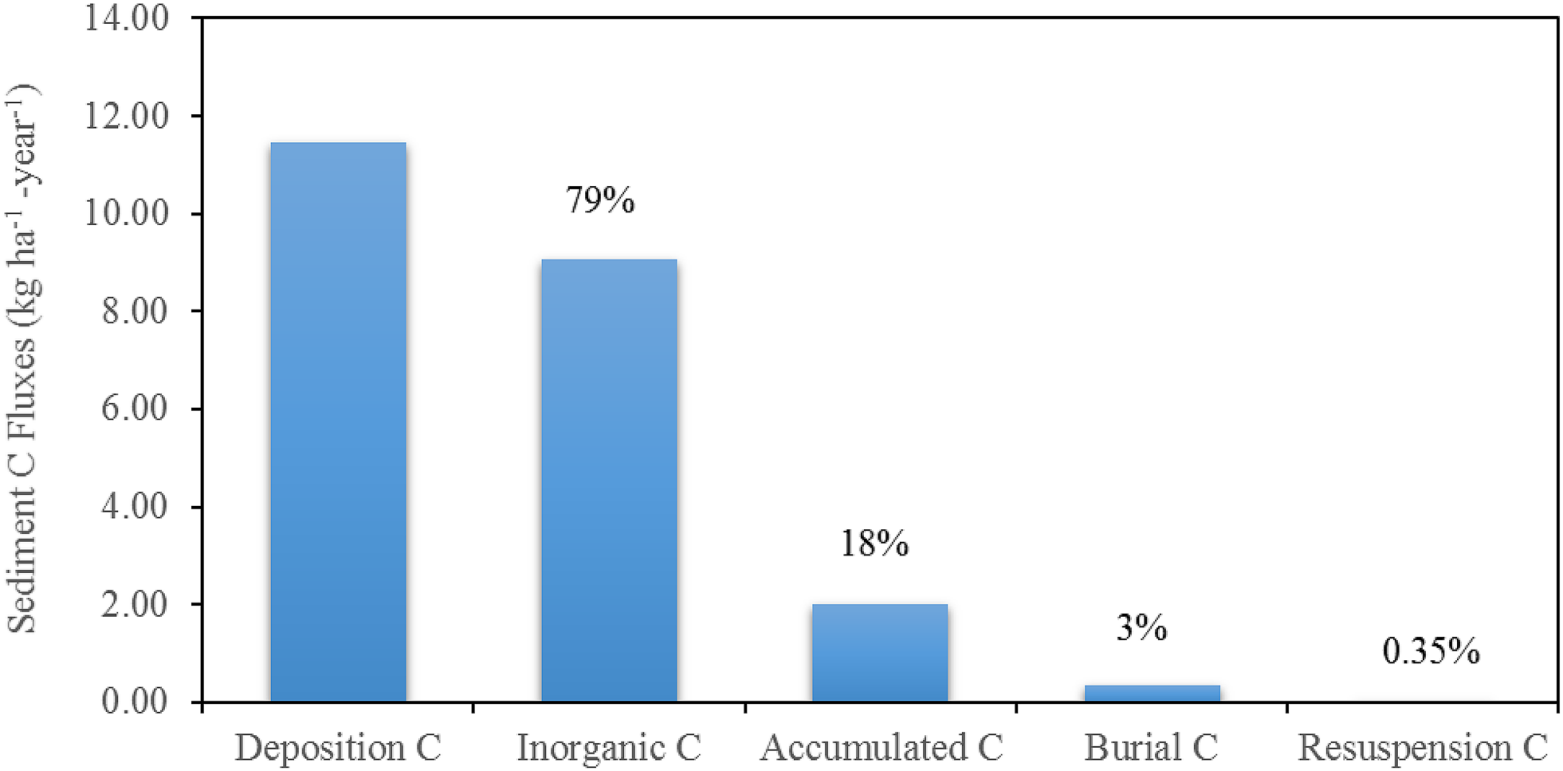
Benthic carbon cycle budget for the TW from 2014 to 2017. The figure also includes percentage of inorganic C (CO_2_ + CH_4_), burial C, resuspension C, and accumulated C fluxes in the total deposited organic C flux in TW

To illustrate the importance of benthic carbon cycle processes, we summarized major components of sediment C fluxes over the riverbed in the TW as shown in Fig. 5, including total organic C deposited on the river bed, resuspension C, burial C, and total inorganic C lost from the sediment (equal to CH_4_ + CO_2_ through diffusion and bubbling processes). The percentages of resuspended C, burial C, total inorganic C lost, and accumulated sediment C in total organic C deposited on the riverbed (%) are also shown in Fig. 6. In general, total organic C (including terrestrial POC and algae debris) deposited on sediment bed was about 11.5 kgC ha^−1^ year^−1^ (accounting for about 47% of outflow POC) over the 4 years in the TW. About 0.04 and 0.34 kgC ha^−1^ year^−1^ organic C were resuspended into the overlying water column and buried out of the sediment system, respectively, accounting for 0.35 and 3% of total deposited organic C in the TW. Combined CH_4_ and CO_2_ transport from sediment to the overlying water column was 9.05 kgC ha^−1^ year^−1^ (equal to 0.85 gC m^−2^ day^−1^ over the water surface area) accounting for 79% of total deposited organic C (Fig. 5), indicating that most of deposited organic C were converted into CO_2_ + CH_4_ in the sediment. These results are comparable to numerical simulation results from Di Toro [59] and observations in the Upper Potomac Estuary [59, 96]. By subtracting C losses from the total deposited C in the sediment, about 2.02 kgC ha^−1^ year^−1^ (accumulated sediment C) remained on the riverbed, which accounted for 18% of total deposited organic C. Simulation results indicate that a large portion of deposited organic C was converted to inorganic C in the sediment and released into the overlying water column. The release of CO_2_ and CH_4_ fluxes will result in elevated concentration of CO_2_ and CH_4_ in the water column and critical sources to atmospheric greenhouse gases. Quantification of CO_2_ and CH_4_ outgassing from inland waters is an important step to fill the gaps in the C budget at the regional or global scale. The result demonstrates the importance of quantification of sediment fluxes for constraining C cycles in aquatic ecosystems and sediment diagenesis processes in water quality modeling. Future development of SWAT-C will include CH_4_ and CO_2_ transformation and transfer processes in the water column and at the water–air interface.

## Conclusion

This study developed a sediment diagenesis and sediment carbon (C) resuspension module within the framework of the SWAT-C model to improve the simulation of riverine organic C cycling. The new version of SWAT-C was tested for simulating both particulate organic C (POC) and dissolved organic C (DOC) fluxes against 4 years of monthly observations (2014–2017) in a small watershed, i.e., the Tuckahoe watershed (TW) in the U.S. Mid-Atlantic region. Evaluated with the two statistical metrics (i.e., percent bias and the Nash–Sutcliffe coefficient), the SWAT-C model satisfactorily simulated monthly POC and DOC fluxes during both calibration and validation. We also conducted sensitivity analyses for parameters that regulate POC settling and sediment C resuspension. The results show that POC deposition parameters tended to be more sensitive than parameters associated with C resuspension, suggesting that POC settling processes were the main factors controlling POC fate in the study watershed.

Based on simulation results, we summarized and analyzed average annual POC and DOC, and fluxes from terrestrial to aquatic environments (including fluxes from uplands, buried in sediments, outflow at the outlet, and deposition/resuspension) in the TW. We found that annual POC and DOC fluxes were about 36.6 and 46 kgC ha^−1^ from allochthonous sources and both were less than 0.72 kgC ha^−1^ from autochthonous sources (< 2% of allochthonous sources) in the river system. Simulation results show that net deposition (equal to deposition–resuspension) of POC on the sediment bed was 11.4 kgC ha^−1^ year^−1^ which accounts for 31% of total POC coming from uplands. In addition, average annual buried C in the sediment bed of the watershed was 0.34 kgC ha^−1^ year^−1^ accounting for 1% of POC fluxes from uplands and 3% of net POC deposition. We further quantified sediment C fluxes by summarizing inputs to, and losses from, the sediment in the TW. Results indicate that a large portion (about 79%) of deposited organic C was converted to inorganic C (CH_4_ and CO_2_) in the sediment and eventually released into the overlying water column. We also confirmed that there is considerable uncertainty regarding estimation of buried C at the watershed scale due to the large range of burial velocities reported in inland waters and coastal environments.

This study serves as an exploratory study on estimation of C fluxes from terrestrial to aquatic environments at the watershed scale. We demonstrated capabilities of the SWAT-C model to simulate C cycling from uplands to riverine ecosystems and simulation results showed that the newly developed SWAT-C model can be used to simulate C sinks and sources in aquatic environments. Given the importance of sediment diagenesis processes, the results point to the need of future development of the SWAT-C model to includes a water column CH_4_ module, gas aeration (CO_2_, CH_4_, and O_2_) algorithms, and a total inorganic C cycling module. The SWAT-C model will be a useful tool to inform C related ecosystem services for watershed assessment and planning.

## Data Availability

The observed riverine particulate and dissolved organic carbon data are provided in Fig. 3.

## Abbreviations

ADJ_PKR: Peak rate adjustment factor in tributary channels
ALPHA_BF: Baseflow alpha factor
C: Carbon
CBP: Chesapeake Bay Program
CDL: Cropland Data Layer
CN2: Curve number
CRW: Choptank River Watershed
DOC: Dissolved organic carbon
ESCO: Soil evaporation compensation factor
GW_DELAY: Groundwater delay
NASA: National Astronautics and Space Administration
NASS: National Agriculture Statistics Service
NLDAS2: North-American Land Data Assimilation System 2
NRCS: Natural Resources Conservation Service
POC: Particulate Organic Carbon
PRF: Peak rate adjustment factor in main channels
SFTMP: Snowfall temperature
SLSOIL: Slope length for lateral flow
SOD: Sediment oxygen demand
SPCON: Linear parameter for sediment routing in main channels
SPEXP: Exponent parameter for sediment routing in main channels
SSURGO: Survey Geographic Database
SURLAG: Surface runoff lag coefficient
SWAT: Soil and Water Assessment Tool
TIC: Total inorganic carbon
TW: Tuckahoe watershed
USDA: United States Department of Agriculture
USEPA: United States Environmental Protection Agency
USGS: United States Geological Survey
USLE: Universal soil loss equation
